# Dapagliflozin’s Effects on Urinary Albumin and Non-Albumin Proteins in Diabetic and Non-Diabetic Kidney Transplant Recipients

**DOI:** 10.3390/biomedicines13061303

**Published:** 2025-05-26

**Authors:** Giancarlo Bilancio, Sarah Hamzeh, Nicoletta Vecchione, Dora Russo, Candida Iacuzzo, Luca Apicella, Renata Angela Di Pietro, Piercarla Vitale, Maria Amicone, Antonio Pisani, Massimo Cirillo, Carmine Secondulfo

**Affiliations:** 1 Department of Medicine, Surgery and Dentistry “Scuola Medica Salernitana”, University of Salerno, 84081 Baronissi, Italy; pievitale@unisa.it (P.V.); mcirillo@unisa.it (M.C.); csecondulfo@unisa.it (C.S.); 2 Department of Public Health, University of Naples “Federico II”, 80131 Naples, Italy; sarahhamzeh1@gmail.com (S.H.); nicolettavecchione@gmail.com (N.V.); dora.russo94@gmail.com (D.R.); ma.amicone.90@gmail.com (M.A.); antonio.pisani13@gmail.com (A.P.); 3 Unit of Nephrology, Dialysis and Transplant, Salerno University Hospital “San Giovanni di Dio e Ruggi d’Aragona”, 84131 Salerno, Italy; candida.iacuzzo@sangiovannieruggi.it (C.I.); luca.apicella@sangiovannieruggi.it (L.A.); angela.dipietro@sangiovannieruggi.it (R.A.D.P.)

**Keywords:** albuminuria, non-albumin proteinuria, kidney transplant, SGLT2is

## Abstract

**Background**: SGLT2 inhibitors (SGLT2is) lower glucose and have renoprotective effects, including reducing proteinuria. In kidney transplant recipients (KTRs), proteinuria impacts graft and patient survival. While SGLT2is benefits have been reported in diabetic KTRs, the data on non-diabetic KTRs are poor, and no data are available for albuminuria and non-albumin proteinuria. This study assessed the effects of dapagliflozin on urinary protein excretion in KTRs with and without diabetes. **Methods**: This analysis, from the Salerno CKD Cohort Study, included 66 KTRs (≥1 year post-transplant) with proteinuria despite renin–angiotensin system inhibitor therapy. The patients received dapagliflozin (10 mg/day) for six months, with assessments at the baseline (T0), three months (T1), and six months (T2); adverse events were monitored. The primary outcomes were changes in the urinary total, albumin, and non-albumin proteins. The secondary outcomes included weight, blood pressure, and eGFR. **Results**: At T1, the urinary total, albumin, and non-albumin proteins were significantly decreased, with a greater reduction in the non-albumin proteins vs. albumin (−27% vs. −9.4%, *p* = 0.001). No further changes occurred at T2. The patients’ weight and blood pressure also declined, while their eGFR and glucose remained stable. The non-albumin protein reduction was correlated with weight loss and diastolic blood pressure changes. Two patients discontinued use due to adverse events (one with a urinary tract infection, one with hypotension). **Conclusions**: Dapagliflozin reduces proteinuria, particularly non-albumin proteins, in KTRs with and without diabetes, with a low incidence of adverse effects. Further studies are needed to confirm the long-term benefits, especially in non-diabetic recipients.

## 1. Introduction

Chronic kidney disease (CKD) currently has a prevalence of 9.1% among the world’s population, constituting increase of 29.3%, with a mortality rise of 41.5% [1]. The discovery of significant benefits from sodium–glucose cotransporter type 2 inhibitors (SGLT2is), a new class of drugs originally used for the treatment of diabetes mellitus, is therefore an extremely interesting novelty in the treatment of chronic kidney disease (CKD).

Sodium–glucose cotransporter type 2 inhibitors (SGLT2is) are drugs originally proposed for the treatment of diabetes mellitus because they lower serum glucose via increased urinary glucose excretion [2]. Their ability to reduce cardiovascular events and to slow CKD progression in diabetic and non-diabetic renal patients provides a new major therapeutic option for these subjects [3,4,5,6,7,8]. A kidney transplant is currently the best available modality for renal replacement therapy, as it affords superior survival and quality of life at a lower cost compared to dialysis [9]. Thus, there is great interest in further prolonging graft survival in kidney transplant recipients (KTRs). Traditionally, the long-term outcomes of kidney transplants have remained unchanged. Conversely, there has recently been a small but noticeable improvement in graft survival half-lives, now expected to be above 11 years and 19 years for deceased and living kidney donors, respectively [10]. These results are not only due to a better understanding of the immune mechanisms behind graft rejections and the subsequent advances in immunosuppressive therapy, but also to advances in the care of the comorbidities, mainly diabetes and hypertension, that vastly affect both patient and graft survival [10,11]. Moreover, proteinuria is actually considered one of the strongest predictors of graft failure [12], and any progress in controlling this parameter could potentially improve survival. It is important to note that current research primarily focuses on the effects of SGLT2is on albuminuria. However, in kidney transplant recipients, proteinuria is more commonly associated with chronic tubulointerstitial damage. As a result, proteinuria in these patients often includes a substantial proportion of non-albumin proteins [13,14,15]. This distinction suggests that ongoing studies may be overlooking a critical aspect relevant to this specific patient population.

As KTRs were not enrolled in the major clinical trials on SGLT2is, the research data on the antiproteinuric effects of SGLT2is in KTRs with diabetes [16,17,18,19] are scanty, and even more so in KTRs without diabetes [20]. Moreover, the evidence for the adverse effects of SGLT2is in KTRs is controversial; while some studies have suggested that urinary tract infections and euglycemic ketoacidosis are not a big concern of SGLT2is therapy in KTRs [16,18,21], other studies have shown an increase in infectious complications [22,23]. The other beneficial effects on electrolyte metabolism could balance these potential concerns [22,24].

Further research is needed on this topic, as the benefits of SGLT2is could potentially be outbalanced by infective complications, which represent a key concern for KTRs in the setting of immunosuppression. This study aims to fill this critical knowledge gap by evaluating the efficacy, especially concerning proteinuria reduction in both albumin and non-albumin urinary proteins; safety; and tolerability of SGLT2is therapy in KTRs subjects, encompassing both diabetic and non-diabetic populations.

## 2. Methods

This analysis is part of the “Salerno CKD Cohort Study”, an ongoing, open-ended, observational study on the whole spectrum of chronic kidney diseases, including KTRs [25]. The standard care for KTR patients covered by the Italian National Health Service includes 3-4 out-patient visits per year with lab work-ups and hospitalization if needed, regardless of where the transplantation was originally performed.

Inclusion criteria for this analysis were age ≥ 18 years, KTR age ≥ 1 year, proteinuria while on treatment for at least 12 weeks with renin–angiotensin system inhibitors at the maximum tolerated dose, and eGFR ≥ 25 mL/min/1.73 m^2^, per requirements of the Italian Drug Agency [26]. Proteinuria was defined as urinary protein–creatinine ratio ≥ 200 mg/g and/or urinary albumin–creatinine ratio ≥ 30 mg/g [27]. Given that treatment with SGLT2is can increase the risk of urinary tract infections [28], patients were excluded if they had had recurrent incidences of urinary tract infections, defined as 2 episodes of acute bacterial cystitis within the last 6 months, or 3 episodes within the previous year [29]. Enrollable patients were prescribed 10 mg/day of dapagliflozin on top of their ongoing treatment, and underwent clinical and lab assessments before the treatment (baseline, T0) and after three (T1) and six (T2) months of treatment. In addition to total proteins, albumin, and creatinine in 24 h urine, the data collection included sex, patient age, transplant age at enrollment, body weight, blood pressure in the sitting position, ongoing treatments against hypertension and diabetes, serum creatinine, and serum glucose. Regarding adverse events, euglycemic ketoacidosis; hypoglycemia; hypotension, defined as systolic blood pressure < 90 mmHg; and urogenital infections were monitored at each visit. Patients were advised to promptly contact the unit in the event of any new symptoms, with special focus on urinary discomfort or pain, urogenital erythema, dizziness, and asthenia. Automated biochemistry was used for lab measurements of serum and urine, and was performed in in-house facility. 

Urinary non-albumin proteins were calculated as urinary total proteins minus urinary albumin. Urinary proteins were expressed as mg/24 h and as ratio to urinary creatinine. Serum creatinine was calibrated with IDMS-traceable standard [30]; gender and age were used for the calculation of the estimated glomerular filtration rate (eGFR) by the CKD-Epi equation [31]. Diabetes was defined as requiring ongoing antidiabetic drug therapy, fasting plasma glucose ≥ 126 mg/dL, or glycated hemoglobin (A1C) ≥ 6.5% (≥48 mmol/mol) [32]. 

Descriptive statistics were reported as prevalence of categorical variables, mean ± SD of non-skewed numerical variables, and median with inter-quartile range (IQR) of numerical skewed variables (skewness > 2). The statistical analyses included the t-test for numerical non-skewed variables, the Wilcoxon and Mann–Whitney test for numerical skewed variables, and Spearman’s rank correlation for correlation analyses. Results were considered statistically significant for *p* values ≤ 0.05. Statistical analyses were performed using SPSS 19 (IBM, Armonk, NY, USA). Plots were generated using GraphPad Prism 5 (GraphPad Software Inc., San Diego, CA, USA) and R 4.2.1.

## 3. Results

A total of 66 patients met the inclusion criteria for this analysis. The baseline descriptive statistics are shown in Table 1.

The majority of the patients were men, non-obese, and non-diabetic. The 3-month changes in the urinary proteins from the baseline were a significant reduction in the urinary total proteins, urinary albumin, and urinary non-albumin proteins, with the data expressed either as mg/24 h or as mg/g of creatinine; no further significant effects on the urinary proteins were found after 6 months of therapy with dapagliflozin (Table 2). With the changes expressed as a percent of the baseline, the reduction in the urinary non-albumin proteins was greater than the reduction in urinary albumin (−27% vs. −9.4%, *p* = 0.001, Wilcoxon for paired data).

Regarding the other clinical and laboratory variables, the 3-month changes also included a significant reduction in body weight and blood pressure, while they were not significant for the eGFR and serum glucose; similarly to the proteinuria, no further significant changes were found after 6 months of therapy with dapagliflozin, with the only exception being a reduction in body weight (Table 3).

**Figure 1:**
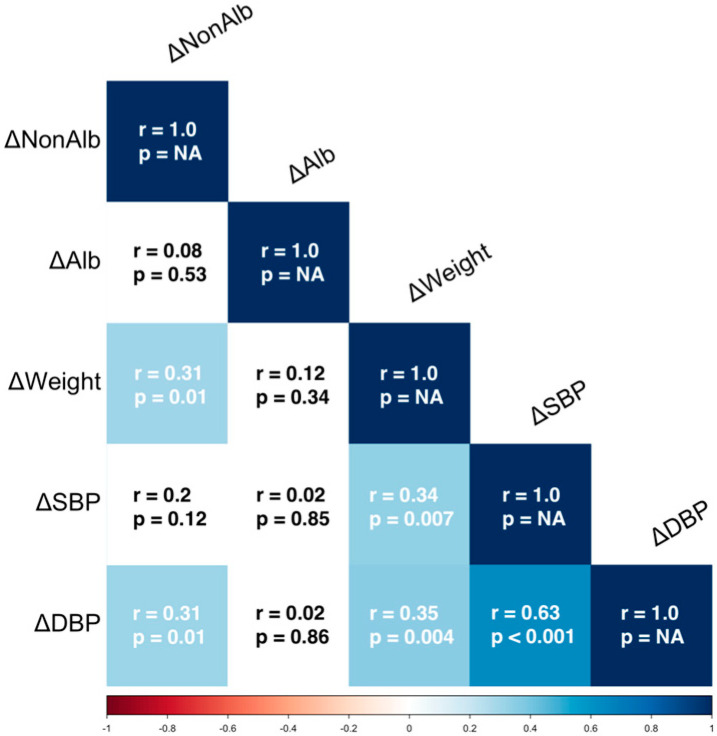
Correlation matrix for changes (Δ) between T1 and T0 in clinical parameters and urinary proteins. Lighter colors indicate weaker correlations, darker colors represent stronger correlations. ΔNonAlb = Changes of urinary non Albumin Proteinuria, ΔAlb = Changes of urinary Albumin Proteinuria; ΔWeight = Changes of Weight; ΔSBP = Changes of Systolic Blood Pressure; ΔDBP = Changes of Diastolic Blood Pressure; *r* = Spearman’s rank correlation coefficient, *p* = *p*-value (statistical significance).

The 3-month changes in diastolic blood pressure were correlated with the non-albumin proteins changes (rank R = 0.314, *p* = 0.012), but were not correlated with changes in urinary albumin (R = −0.022, *p* = 0.866); the changes in systolic blood pressure were not correlated with the urinary albumin (R = −0.025, *p* = 0.848) or urinary non-albumin proteins (R = 0.20, *p* = 0.116). The 3-month change in body weight was correlated only with a reduction in the non-albumin proteinuria (R = 0.307, *p* = 0.014), while no correlation was found with albuminuria (R = −0.121, *p* = 0.340) (Figure 1).

Two patients presented with adverse effects after the first week of treatment: one subject had a urinary tract infection and another presented with hypotension. The SGLT2is administration was promptly withdrawn. Due to the early interruption of their therapy with dapagliflozin, the data from these patients were not available at either T1 or T2, and were thus excluded from the analysis. Ketoacidosis and hypoglycemia were not found in any of the patients.

**Figure 2:**
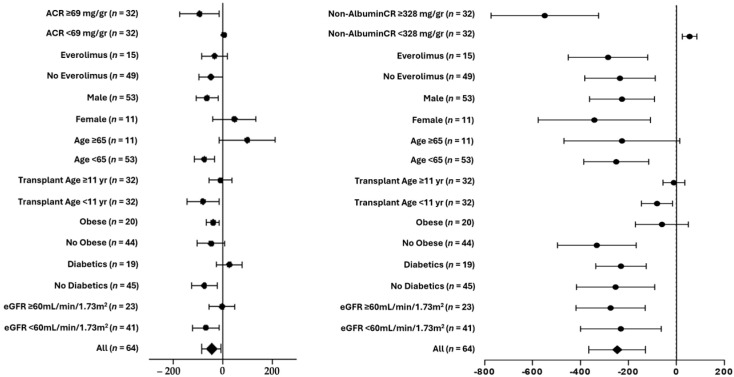
Subgroup analyses of 3-month changes in delta mean (SE) of urinary albumin/creatinine (left panel) and non-albumin/creatinine (right panel).

The subgroup analyses in Figure 2 show that the effects on the urinary albumin and non-albumin proteins were similar for males and females, immunosuppressive therapy with or without everolimus, ages ≥ 65 years and ages < 65 years, transplant age > 11 years or ≤11 years, obese and non-obese patients, diabetic and non-diabetic patients, and an eGFR ≥ 60 mL/min/1.73 m^2^ and < 60 mL/min/1.73 m^2^. A significant difference was found only for the delta of the non-albumin/creatinine ratio for subjects with a non-albumin/creatinine ratio above or below the median of the cohort.

## 4. Discussion

The present study demonstrates that a three-month treatment with dapagliflozin led to a reduction in both urinary albumin and non-albumin protein excretion, as well as decreases in body weight and blood pressure. Importantly, these beneficial effects occurred without any significant changes in serum glucose levels or the estimated glomerular filtration rate (eGFR), indicating a favorable safety profile with respect to glycemic control and renal function. The incidence of adverse events was low: only one patient experienced asymptomatic hypotension, while another developed symptomatic hypotension. Notably, no cases of euglycemic ketoacidosis or hypoglycemia were observed among the study participants. The observed outcomes were consistent across the subgroup analyses and remained stable in those patients who continued therapy for a total duration of six months, further supporting the treatment’s efficacy and tolerability over time.

The present findings confirm and extend previous reports on gliflozin’s effects in KTRs with diabetes, as in the studies of Attallah et al. and Sheu et al. [33,34], and in those without diabetes, as in the studies by Maigret et al. and Quilis et al. [20,35]. This study, however, further analyzed the different impacts on albumin and non-albumin urinary proteins, which is unreported in the current literature.

The lack of significant changes in the mean estimated glomerular filtration rate (eGFR) following three and six months of treatment with dapagliflozin aligns with the findings from two previously published studies [36,37]. This observation is also consistent with prior evidence indicating that any reduction in the glomerular filtration rate associated with SGLT2 inhibitor therapy tends to be transient and typically occurs within the first month of treatment only [16]. These findings further confirm that, beyond the initial adjustment period, dapagliflozin does not exert a detrimental effect on the overall renal function of kidney transplant recipients, similarly to the general kidney patient population.

Given that urinary non-albumin proteins are generally considered a marker of tubulointerstitial dysfunction [13], the baseline data on the urinary non-albumin proteins support the view that proteinuria in kidney transplant patients also reflects tubulointerstitial damage [14,15,38]. The post-treatment changes in the urinary non-albumin proteins suggest the intriguing possibility of dapagliflozin’s effects on the handling of protein at the level of the kidney tubule, given that the reduction in the urinary non-albumin was significant and actually greater than the reduction in the urinary albumin. The current literature suggests that the renoprotective properties of SGLT2 inhibitors on renal proximal tubular epithelial cells (RPTECs) may be partially mediated by hemodynamic mechanisms and by protection from glucotoxic stress [39,40]. Other mechanism(s) cannot be excluded. This finding suggests that the beneficial effects of SGLT2 inhibitors may play a valuable role in mitigating the consequences of tubulointerstitial damage. Given that this form of injury is a prominent contributor to long-term graft dysfunction in kidney transplant recipients, and that effective therapeutic strategies targeting this process remain limited, the potential of SGLT2 inhibitors in this context represents an area of significant clinical interest. These preliminary observations highlight the need for further investigations into the mechanisms by which SGLT2 inhibitors may influence tubulointerstitial pathology, an aspect of post-transplant care that is currently underexplored and largely unmet. According to the current views [33], the antiproteinuric effects of gliflozin are regarded as due to changes in blood pressure or hemodynamics. The present results are in partial agreement with this view, because the reduction in urinary non-albumin proteins was correlated with a diastolic blood pressure reduction, while the urinary albumin reduction was not.

The weight loss observed during SGLT2is therapy aligns with the existing literature [41], and the correlation between weight loss and a reduction in proteinuria is well established [42,43]. However, our findings indicate that these effects may be more pronounced for non-albumin proteinuria in kidney transplant recipients—an observation that, to our knowledge, has not been previously reported.

This study’s limitations are the uncontrolled study design; the number of patients; the enrollment of patients from a single center only; and the 6-month duration, which might have underscored the untoward side effects. Moreover, a minority of the patients included in the cohort were treated with everolimus, which is known for its adverse effects on proteinuria [44]. Another potential limitation of this study is the absence of data on the primary kidney disease in 40% of the patient cohort, as well as the lack of precise information regarding the underlying cause of proteinuria. Among its possible merits, it is the first report on non-diabetic KTRs and on urinary non-albumin proteins.

In conclusion, dapagliflozin reduced proteinuria—both albumin and, as a novelty, non-albumin proteins—in KTR patients with diabetes and without diabetes, with a low rate of side effects. A greater effect was observed on the non-albumin urinary proteins, especially in the subjects with higher non-albumin proteinuria. In some cases, the magnitude of the observed changes was relatively small despite the statistical significance. However, given the fundamental prognostic role of proteinuria in graft survival and patient survival, any possible amelioration is clinically relevant.

Long term-controlled studies will be needed to verify if this treatment is also effective for the prevention of severe complications, especially for non-diabetic transplant recipients.

## Figures and Tables

**Table 1:** Baseline characteristics: mean ± SD for non-skewed data and median (IQR) for skewed data at the time of gliflozin’s first administration.

**Number of patients**	64
**Men, %**	82.8%
**Age, years**	55.1 (41.8–62.1)
**Primary Kidney Disease**	
Unknown, %	40.6%
Glomerular, %	35.9%
Genetic, %	10.9%
Interstitial, %	1.6%
Metabolic, %	1.6%
Other, %	9.4%
**Immunosuppressive Therapy**	
Cyclosporine, %	31.3%
Tacrolimus, %	64.1%
Everolimus, %	23.5%
Mycophenolate, %	70.3%
Steroid, %	95.3%
**Transplant Age, years**	11.1 ± 8.4
**Obesity, %**	31.3%
**Diabetes, %**	29.7%
PTDM, % (of diabetics)	52.6%
Impaired Fasting Glucose	12.5%

PTDM = Post-Transplant Diabetes Mellitus.

**Table 2:** Baseline (T0), 3 (T1)-, and 6 (T2)-month medians (IQRs) of urinary variables.

	T0 (*n* = 64)	T1 (*n* = 64)	T2 (*n* = 64)
Urinary protein/creatinine, mg/g	383 (188–1461)	^†^ 376 (148–940) **	^‡^ 418 (211–778) ^ns^
Urinary albumin/creatinine, mg/g	69 (13–385)	^†^ 45 (12–252) *	^‡^ 64 (14–214) ^ns^
Urinary non-albumin/creatinine, mg/g	328 (145–945)	^†^ 260 (127–615) *	^‡^ 308 (160–627) ^ns^
24 h urinary proteins, mg	601 (243–1860)	^†^ 472 (200–1209) ***	^‡^ 492 (216–1078) ^ns^
24 h urinary albumin, mg	97 (15–449)	^†^ 59 (12–290) *	^‡^ 60 (14–316) ^ns^
24 h urinary non-albumin proteins, mg	520 (178–1200)	^†^ 344 (176–888) ***	^‡^ 389 (164–842) ^ns^

*n* = number of patients; * *p* < 0.05; ** *p* < 0.005; *** *p* < 0.001; ns = not significative. ^†^ T0 vs. T1; ^‡^ T1 vs. T2 for Wilcoxon paired data.

**Table 3:** Baseline (T0), 3 (T1)-, and 6 (T2)-month means ± SD of clinical and biochemical variables.

	T0 (*n* = 64)	T1 (*n* = 64)	T2 (*n* = 64)
Body weight, kg	81 ± 16	^†^ 80 ± 16 *	^‡^ 80 ± 15 *
Systolic blood pressure, mmHg	139 ± 15	^†^ 133 ± 13 **	^‡^ 134 ± 16 ^ns^
Diastolic blood pressure, mmHg	83 ± 10	^†^ 80 ± 9 **	^‡^ 78 ± 9 ^ns^
Serum creatinine, mg/dL	1.47 ± 0.45	^†^ 1.54 ± 0.52 *	^‡^ 1.6 ± 0.60 *
eGFR, mL/min/1.73 m^2^	56 ± 18	^†^ 53 ± 19 ^ns^	^‡^ 52 ± 20 ^ns^
Serum glucose, mg/dL	108 ± 18	^†^ 103 ± 25 ^ns^	^‡^ 106 ± 28 ^ns^

*n =* number of patients; * *p* < 0.05; ** *p* < 0.005; ns = not significant. ^†^ T0 vs. T1; ^‡^ T1 vs. T2 for *t*-test for paired data.

## Data Availability

The data underlying this article will be shared upon reasonable request to the corresponding author.
